# Substance use and sexual behaviours of Japanese men who have sex with men: A nationwide internet survey conducted in Japan

**DOI:** 10.1186/1471-2458-6-239

**Published:** 2006-09-26

**Authors:** Yasuharu Hidaka, Seiichi Ichikawa, Junko Koyano, Michiko Urao, Toshihiko Yasuo, Hirokazu Kimura, Masako Ono-Kihara, Masahiro Kihara

**Affiliations:** 1 Department of Global Health and Socio-epidemiology, Kyoto University School of Public Health Yoshidakonoe-cho, Sakyo-ku, Kyoto 606-8501, Japan; 2 Japanese Foundation for AIDS Prevention 5th floor, 1-3-12, Misaki-cho, Chiyoda-ku, Tokyo 101-0061, Japan; 3 Nagoya City University School of Nursing 1 Kawasumi, Mizuho-cho, Mizuho-ku, Nagoya 467-8601, Japan; 4 Matsuhama Hospital 3396 Matsuhama-cho, Niigata, 950-3121, Japan; 5 Genetic Counselling and Clinical Research Unit, Kyoto University School of Public Health Yoshidakonoe-cho, Sakyo-ku, Kyoto 606-8501, Japan; 6 AIDS Medical Center, Osaka National Hospital 2-1-14, Hoenzaka, Chuo-ku, Osaka, Japan; 7 Minami Public Health and Welfare Center, City of Yokohama 3-48-1 Hananogi-cho, Minami-ku, Yokohama, 232-0018, Japan

## Abstract

**Background:**

Japanese men who have sex with men (MSM), especially those living in large metropolitan areas such as Tokyo and Osaka, are facing a growing HIV/AIDS epidemic. Although the Internet is used as a new venue for meeting sex partners, it can also serve as a useful research tool for investigating the risk behaviours of Japanese MSM. This Internet survey explored the extent of substance use and its association with sexual risk behaviours among Japanese MSM.

**Methods:**

Between 28 February 2003 and 16 May 2003 MSM were recruited through 57 Japanese gay-oriented Web sites, gay magazines, and Internet mailing lists. Participants completed a structured questionnaire anonymously through the Internet.

**Results:**

In total, 2,062 Japanese MSM completed the questionnaire. The average age of participants was 29.0 years and 70.5% identified as gay, 20.8% as bisexual, and 8.7% as other. Overall, 34.5% reported never using a substance, 45% reported ever using one type of substance (lifetime reported single substance users), and 19.6% had used more than 1 type of substance (lifetime reported multiple substance users) in their lifetimes. The substances most commonly used were amyl nitrite (63.2%), 5-methoxy-N, N-diisopropyltryptamine (5MEO-DIPT) (9.3%), and marijuana (5.7%). In the multivariate analysis, unprotected anal intercourse, having had 6 or more sexual partners, visiting a sex club/gay venue in the previous 6 months, a lower education level, and being 30 to 39 years of age were associated with both lifetime single and lifetime multiple substance use. Lifetime reported multiple substance use was also correlated with having a casual sex partner, having symptoms of depression, being diagnosed as HIV-positive, and greater HIV/AIDS-related knowledge.

**Conclusion:**

This is the first Internet-based research focused on the sexual and substance use behaviours of MSM in Asia. Our findings suggest a compelling need for prevention interventions to reduce HIV risk-related substance use behaviours among Japanese MSM. The results also suggest that the Internet is potentially a useful tool for collecting behavioural data and promoting prevention interventions among this population.

## Background

Recently, the HIV epidemic has spread rapidly among men who have sex with men (MSM) in Japan. New HIV infections through sexual contact among MSM have continued to rise steadily over the last two decades with data indicating the rate of increase accelerating since 1999. According to the 2005 annual surveillance report on HIV/AIDS by the AIDS Surveillance Committee of the Ministry of Health, Labour, and Welfare, there were 7,392 people infected with HIV and 3,644 people with AIDS. In addition, there were 1,435 people who acquired with HIV/AIDS through unheated blood products. In 2005, Japanese MSM accounted for 66.8% of new HIV infections and 37.9% of new AIDS cases reported among men [1]. Of new HIV infections reported among MSM, the majority were from metropolitan areas such as Tokyo and Osaka, indicating that these are potential centres of an emerging HIV epidemic in Japan.

High levels of substance use have been reported among MSM populations in the USA, Australia and substance use is increasing among MSM in Asian countries, such as Nepal and the Philippines [2-10]. Studies suggest that substance use is strongly associated with high-risk sexual behaviours [3,11,12]. There is therefore a pressing need to collect data on the sexual and substance use behaviours of MSM to understand HIV risk and develop effective prevention programmes. However, research on substance use and sexual behaviours among Japanese MSM has been limited due to the deeply rooted social prejudice towards homosexuality and the illegal nature of substance use in Japan.

The use of the Internet is widespread in Japan, with the Ministry of Internal Affairs and Communication White Paper reporting 68% of Japanese households having Internet access in 2005[13]. At the end of 2003, Internet usage rates of 90% were reported in the following age groups: 13 to 19, 20 to 29, and 30 to 39 [14]. The Internet has developed into a useful research tool for conducting surveys and is increasingly used to conduct behavioural surveys of hard-to-reach or hidden populations, such as MSM [15]. The Internet enables researchers to engage with anonymous participants, allowing access to populations that cannot be reached using conventional sampling procedures, such as venue-based or snowballing samplings. In addition, it allows the collection of a large number of participants in a short period of time, beyond geographic boundaries, with lower costs. The Internet has also been used increasingly as an effective tool for general and personalized intervention because of its universal accessibility and interactive nature.

Many Japanese MSM use the Internet and it has become a vital tool for meeting new partners and friends, developing relationships, and making sexual contacts [16,17]. Given the social stigma facing this group, the use of the Internet to meet new partners might be especially important for MSM who are not open about their sexuality, those who prefer that their interest in same-sex relationships remain hidden.

This study is the first Internet-based survey in Japan to explore substance use and sexual behaviours among Japanese MSM. The data will be used to inform the development of effective Internet-based health education and HIV prevention programmes for Japanese MSM.

## Method

### Sampling

A Web site was created to host this Internet survey, and sampling was conducted continuously from 28 February to 16 May 2003. In order to attract potential research participants, unpaid and paid banners advertising the research project were posted on 57 gay related Web sites and announcements were published in gay magazines and Internet mailing lists.

To prevent duplicate responses, cookie data were collected to determine the first response from an individual Internet browser. To participate, a male must have had sexual experience(s) with other male(s), be currently residing in Japan, be able to read and write Japanese, and have access to the Internet. In addition, to confirm that the participant was a member of the target group, respondents were asked about the meaning of 2 slang words that are used by Japanese gay men. Individuals who could not answer the questions were deemed ineligible, based on the supposition that they were not MSM and their responses were not included in analysis.

Informed consent was requested from all participants on the first page of the questionnaire, and only those who consented were given access. The study protocol was approved by the Ethics Committee of Kyoto University Graduate School and Faculty of Medicine.

### Measures

The questionnaire was developed from the results of a previous study that focused on psychological and social problems among 388 Japanese MSM who participated in an online qualitative study [18]. The questionnaire was developed in collaboration with a clinical psychologist who had clinical experience with MSM and people living with HIV/AIDS. A pilot survey was conducted with 47 individuals recruited via the Internet to clarify the wording of the questionnaire. Questionnaire items included age; educational background; sexual orientation; existence of a casual male sex partner; HIV status; lifetime histories of hepatitis A, hepatitis B, and syphilis; and HIV antibody test history in the previous year. The questionnaire also assessed depression using the Japanese version of the Self-rated Depression Scale [19] adapted from the original Zung measure [20]; scores above 50.0 have been shown to indicate high levels of depression in Japanese subjects [21] (Cronbach's alpha=.917). Questions were also asked sexual behaviours in the previous 6 months including: unprotected anal intercourse (UAI), the number of male sexual partners, and the frequency of attending gay venues (gay bars) and sex clubs. HIV/STI knowledge was assessed using five true-or-false items: 'You can assess your HIV status 2 to 3 days after a high-risk event'; 'You are more likely to be infected with HIV if you have an STI'; 'You can contract an STI through oral sex'; 'You will not contract an STI through insertive anal sex'; and 'You will not contract HIV through insertive anal sex'. The participants were divided into two groups based on their responses: those who answered all five items correctly, and those who answered one or more question incorrectly.

Questions regarding substance use included lifetime use of the substances listed in Table 1. The reason for asking for lifetime substance use was based on the results of the study's pilot study in which many participants expressed hesitancy in reporting recent involvement in illegal substance use.

### Statistical analysis

Bivariate and multiple logistic regression analyses were conducted to identify correlates of substance use. The subjects were divided into three groups according to their lifetime substance use history: those who never used any substance ("never-used" group), those who used only one type of substance ("lifetime reported single substance user" group), and those who used more than one type of substance ("lifetime reported multiple substance user" group). The never-used group was compared to each substance user group using the chi-square test. Multiple logistic regression analysis was then used to evaluate the independent correlations of demographic, behavioural, and psychological variables with substance use in each substance user group, with the never-used group as the reference. Due to the cross-sectional design and the different time frames of questions regarding substance use and sexual behaviour, causal analysis cannot be made.

## Results

### Demographic characteristics

There were 2,195 respondents to the survey, and the data from 2,062 participants were used. Of the 133 respondents who were eliminated, 88 had incomplete questionnaires and 45 did not live in Japan. The average age of the participants was 29.0 years (range 14–76, SD = 8.0). Sixty percent had university degrees or higher. Regarding sexual orientation, 70.5% identified as gay, 20.8% as bisexual, and 8.7% as other. Of the participants, 73% resided in Tokyo, Osaka, or another urban area.

### Substance use

Of the participants, 65% had lifetime experience of substance use and 35% had never used (see Table 1). The most frequently used substances were amyl nitrite (63.2%), followed by 5-methoxy-N,N-diisopropyltryptamine (5MEO-DIPT) [22] (9.3%), known as "*gomeo*", marijuana (5.7%), and other substances (0.1–3.3%). Among lifetime reported single substance users, the vast majority (96.4%) reported using amyl nitrite. Most of the lifetime reported multiple substance users had used amyl nitrite (96.8%) and almost half had used 5MEO-DIPT. In addition, substance use involving marijuana (25.4%), magic mushrooms (16.8%), Viagra (15.1%), ecstasy (13.8%), and methamphetamine (13.3%) were reported. Of the lifetime reported multiple substance users, only 18 (4.4%) had ever injected substances and one of them was HIV-positive. Overall, 70% of both lifetime reported single and multiple substance users resided in Tokyo, Osaka, or other urban areas.

### Correlates of type of substance and sexual behaviour

Bivariate analyses showed higher percentages of unprotected sexual activities among substance users, particularly among lifetime reported multiple substance users (See Table 2). Regarding sexual behaviour within the previous 6 months, 45% of lifetime reported multiple substance users reported a casual sex partner with another man (male sex friend), 49.6% had four or more sexual partners, 57.0% had unprotected anal intercourse (UAI), 64.2% visited a sex club, and 73.3% visited a gay venue. Lifetime reported multiple substance use was associated with a higher frequency of diagnosis with STIs: HIV 7.4%, syphilis 17.8%, hepatitis A 3.0%, and hepatitis B 10.9%. Older age, less education, HIV testing within the previous year, and higher HIV-related knowledge scores were also concentrated among lifetime reported single and multiple substance users, with a greater concentration among lifetime reported multiple substance users. Depression was associated with substance use only in lifetime reported multiple substance users.

Table 3 shows the results of the multivariate regression analysis conducted to identify independent correlates of substance use inputting all variables compulsorily, except for a history of diagnosis with syphilis or hepatitis which were closely correlated with HIV infection. The analysis revealed that UAI and visiting sex club/gay venues in the previous 6 months were significantly associated with the lifetime reported single and multiple substance user groups and the association was strongest with lifetime reported multiple substance use. The association of having six or more sex partners in the previous 6 months, educational background, and age group showed a consistent pattern across the substance user groups and peaked in the 30 to 39 year age group. Having had an HIV test within the previous year reached statistical significance in both lifetime reported single and multiple substance user groups. Lifetime reported multiple substance use was significantly correlated with having a casual male sex partner, depression, being HIV-positive, and HIV-related knowledge.

## Discussion

As the first Internet survey conducted with a large sample of MSM in Japan, it is the first to reveal the profile of substance use and its relationship with sexual behavior among this population. Our results indicate that amyl nitrite was ever used by 63.2% of respondents, 5MEO-DIPT by 9.3% suggesting that amyl nitrite and 5MEO-DIPT represent the substances most commonly used. Frequent use of 5MEO-DIPT has been also observed in other study conducted among Japanese MSM in 2003 where 18.8% of 576 gay night club clients reported lifetime use of 5MEO-DIPT [23]. Comparison with the results of a randomized nationwide general population survey on substance use, conducted in 2005 in Japan [24], suggests that substance use is relatively high among our respondents in that marijuana use is 4 times, Methylenedioxymethamphetamine use 28 times and methamphetamine use 8 times higher than the general population sample, though no comparable data has been available on amyl nitrite or 5MEO-DIPT. Regarding the substance use profile, it is important to note that the most common substances used were those that were not prohibited by law at the time of the survey, though both 5MEO-DIPT and amyl nitrite became prohibited substances in 2005 and 2006, respectively. Rates of use could be biased due to the underreporting of illegal substance use by respondents, though care was taken to ask lifetime use rather than current use. Thus, actual substance use rates among respondents could actually be higher than the situation revealed in our study. The results of the interviews conducted as a part of the pilot survey pointed to discourses by MSM that 5MEO-DIPT and amyl nitrite were as effective as other illegal substances in increasing sexual sensation and that these substances would be safe because the law would not allow the sale and use of unsafe substances. It is possible that the combination of a number of factors, including the efficacy of these substances, feeling of security in obeying the law and false perception of medical safety, lead to the use of 5MEO-DIPT and amyl nitrite in preference to illegal substances. If this is the case, it is possible that the substance use profile has changed substantially since the recent criminalisation of 5MEO-DIPT and amyl nitrite, and it is an imperative that follow up surveys be conducted to determine this.

### Type of substance use and sexual behaviour

There is a body of research indicating the connection between amyl nitrite use and sexual behaviour, including high risk sexual behaviours such as UAI and multiple sexual partners [3,5,6,25]. Our results were consistent with previous studies, indicating that lifetime reported multiple substance users had a greater number of sex partners and engaged in unprotected sex more frequently, placing them at greater risk for HIV and STIs. In fact, among lifetime reported multiple substance users in this study, infection rates of 7.4% for HIV and 17.8% for syphilis were reported; this HIV prevalence is the highest among any MSM subpopulation ever surveyed to date in Japan. The sexual risks of lifetime reported single substance users were no less alarming, since nearly half had engaged in UAI during the previous 6 months and 2.3% reported infection with HIV and 8.0% with syphilis. Similar high rates of syphilis prevalence have also been reported in recent data of Japanese MSM who attended HIV/STIs-testing programmes for gay men in the Osaka area, where syphilis prevalence was found to be 14.7% in 2000, 15.9% in 2001 and 19.6% in 2002 [26], suggesting the possible emergence of a syphilis epidemic among these groups. Since syphilis increases susceptibility to HIV infection, syphilis prevention and treatment programmes for Japanese MSM should be prioritised. Furthermore, substance use needs to be factored into the development of HIV prevention education programmes for MSM in Japan.

In this study, there was a strong association between lifetime reported multiple substance use and depression, which may imply that participants with depressive symptoms might use substances to cope. However, the explanatory pathways remain unclear due to the cross-sectional survey design. Interventions for Japanese MSM could include the use of Internet technologies to provide referrals to specialists, such as clinical psychologists, psychiatrists, and substance treatment and mental health organizations, to address substance use and depression. There is also a need for education to increase knowledge about the needs and concerns of MSM among Japanese medical doctors, nurses, and other public health professionals because homosexuality and same-sex behaviour are poorly understood within Japan's health sector.

Our results suggest that the Internet is a potentially useful tool for promoting intervention measures for Japanese MSM, as reflected in the successful recruitment of large number of participants our Web site achieved within a short period. In addition to disseminating information to the public at large, the Internet could be used to provide personalized intervention or support for vulnerable and more marginalized populations at risk for HIV, and it is particularly relevant in cultures and settings in which MSM remain highly stigmatized and less visible, such as Japan.

### Limitations

There are several limitations to our study. First, it is impossible to determine whether study participants represent the MSM population as a whole or only MSM using the Internet. We also recognize that there was a sampling bias in terms of the respondents' age and educational background. Second, no causal inference was possible because of the cross-sectional design of the study. Third, substance history referred to lifetime behaviour, whereas the time frame for sexual behaviour related questions was the previous six months. Caution should therefore be exercised in interpreting the observed association between substance use and sexual behaviour as the data does not suggest causal relationships, and it may well be that other, as yet unexamined, variable influence sexual risk behaviour and multiple substance use. It is possible that some of the lifetime reported single or multiple substance users were no longer using during the preceding 6 months, which could have weakened the association between lifetime substance use and sexual behaviour. Finally, although the study was conducted through the Internet, there could still be underreporting of sensitive questions, such as HIV status or illicit-substance use.

Future research should specify the time frames for substance use, why and how substances are used by Japanese MSM. Indeed, it is a necessity to clarify the motivation, situational context, and psychological problems associated with substance use among Japanese MSM in order to develop effective education and prevention programmes.

## Conclusion

This is the first academic study to use the Internet to examine the sexual and substance use behaviours of MSM in Asia. Our findings clearly indicate that substance use was widespread among respondents, as was unsafe sexual behaviours and HIV/STIs infection, especially among lifetime reported multiple substance users. These results indicate an urgent need to introduce effective community-based prevention measures for HIV and STIs among MSM in Japan. The present study also suggests that it may be possible to offer prevention programmes via the Internet.

## Competing interests

The author(s) declare that they have no competing interests.

## Authors' contributions

YH conceived this study and developed the overall procedure used in this project, including the questionnaire, sampling, and statistical analysis, and drafted the manuscript. JK, MU, TY and M O-K were responsible for the study design and creating the questionnaire.

SI, HK and MK were responsible for the data analysis and involved in writing and revising the manuscript. All of the authors participated in the study design, and read and approved the final manuscript.

## Pre-publication history

The pre-publication history for this paper can be accessed here:



## Figures and Tables

**Table 1:** Substance use among Japanese MSM recruited through the Internet (N = 2,062).

	Overall n = 2062	Lifetime reported single substance user n = 945	Lifetime reported multiple substance user n = 405
	%	%	%

Amyl nitrites (Poppers)	63.2	96.4	96.8
5-methoxy-N,N-diisopropyltryptamine (5MEO-DIPT)	9.3	0.2	46.9
Marijuana	5.7	1.6	25.4
Magic mushrooms	3.3	0.1	16.8
Viagra	3.1	0.3	15.1
Methylenedioxymethamphetamine (Ecstasy)	2.8	0.2	13.8
Methamphetamine	2.6	0	13.3
Psychotropic agents	2.6	0.6	11.9
Thinner	1.6	0.1	7.7
Crack or Cocaine	0.8	0	4.0
Lysergic acid diethylamide(LSD)	0.6	0	3.0
Heroin	0.2	0	1.5
Injectable steroids	0.1	0	0.7

**Table 2:** Socio-demographics, risk characteristics, and sexually transmitted infections of Japanese MSM recruited through the Internet

		Overall n = 2062	Never users n = 712	Lifetime reported single substance user n = 945		Lifetime reported multiple substance user n = 405	
					
		%	%	%	*P *value	%	*P *value
Age group	14–19	7.8	11.5	6.1	.000	5.5	.000
	20–29	51.4	52.0	53.2		48.3	
	30–39	30.6	24.3	32.8		37.8	
	40+	9.5	12.2	8		8.5	
Education level	No University degree	40.2	36.5	40.5	.103	45.9	.002
	University degree	59.8	63.5	59.5		54.1	
Sexual orientation	Gay	70.5	62.8	75.2	.000	73.1	.002
	Bisexual	20.8	25.7	18.2		18	
	Other	8.7	11.5	6.6		8.9	
Currently have a casual sex partner	No	70.0	78.7	69.9	.000	54.8	.000
	Yes	30.0	21.3	30.1		45.2	
Depression^1^	Low	81.7	84.3	83.4	.638	73.3	.000
	High	18.3	15.7	16.6		26.7	
HIV status	Negative	97.2	99.3	97.7	.010	92.6	.000
	Positive	2.8	0.7	2.3		7.4	
Syphilis	No	92.3	98.5	92.0	.000	82.2	.000
	Yes	7.7	1.5	8.0		17.8	
Hepatitis A	No	98.7	99.2	99.2	1.00	97.0	.011
	Yes	1.3	0.8	0.8		3.0	
Hepatitis B	No	95.1	98.6	95.0	.000	89.1	.000
	Yes	4.9	1.4	5.0		10.9	
UAI in the previous 6 month	No anal intercourse	56.6	18.8	7.1	.000	4.7	.000
	Protected	21.3	49.4	44.3		38.3	
	Unprotected	22.1	31.7	48.6		57.0	
Number of sexual partners in the previous 6 months	0	18.9	26.7	15.7	.000	12.6	.000
	1	23.0	30.1	20.1		17.3	
	2–3	23.7	24.0	24.9		20.5	
	4–5	14.7	9.8	17.6		16.5	
	6+	19.7	9.4	21.8		33.1	
Sex club in the previous 6 months	No	52.0	69.0	46.2	.000	35.8	.000
	Yes	48.0	31.0	53.8		64.2	
Gay venue in the previous 6 months	No	39.6	54.1	34.2	.000	26.7	.000
	Yes	60.4	45.9	65.8		73.3	
Tested for HIV within the past year	No	76.3	85.5	74.1	.000	65.4	.000
	Yes	23.7	14.5	25.9		34.6	
HIV/STI knowledge score	0–4	53.7	61.8	51.6	.000	44.2	.000
	Full score	46.3	38.2	48.4		55.8	

1 Dichotomous categories based on Kawano, Suematsu & Shinzato (1990)

**Table 3:** Multivariate analysis of substance use of Japanese MSM recruited through the Internet.

		Lifetime reported single substance user (n = 945)			Lifetime reported multiple sbstance user (n = 405)		
			
		AOR	95% C.I.	*P*-value	AOR	95% C.I.	*P*-value
Age group	14–19	1			1		
	20–29	1.60	1.07–2.40	.023	1.53	0.85–2.77	.160
	30–39	2.12	1.38–3.25	.001	2.62	1.41–4.86	.002
	40+	1.02	0.62–1.67	.944	1.16	0.57–2.38	.679
Education level	No University degree	1			1		
	University degree	0.74	0.56–0.94	.012	0.66	0.48–0.89	.007
Sexual orientation	Gay	1			1		
	Bisexual	0.81	0.62–1.06	.120	1.00	0.68–1.46	.985
	Other	0.62	0.42–0.92	.016	1.19	0.72–1.98	.502
Currently have a casual sex partner	No	1			1		
	Yes	1.24	0.96–1.60	.104	2.57	1.84–3.59	.000
Depression	No	1			1		
	Yes	1.12	0.84–1.50	.447	2.27	1.58–3.27	.000
HIV status	Negative	1			1		
	Positive	2.91	0.92–9.21	.070	7.78	2.33–25.93	.001
UAI in the previous 6 months	No anal intercourse	1			1		
	Protected	1.57	0.95–2.58	.078	1.20	0.56–2.56	.634
	Unprotected	2.53	1.53–4.17	.000	2.42	1.14–5.16	.022
Number of sex partners in the previous 6 months	0	1			1		
	1	0.82	0.54–1.25	.360	1.08	0.59–1.98	.804
	2–3	1.04	0.68–1.58	.864	1.06	0.59–1.92	.838
	4–5	1.44	0.90–2.29	.127	1.22	0.65–2.30	.543
	6+	1.67	1.05–2.67	.031	2.13	1.15–3.95	.016
Sex club in the previous 6 months	No	1.00			1		
	Yes	1.64	1.29–2.09	.000	2.00	1.44–2.80	.000
Gay venue in the previous 6 months	No	1.00			1		
	Yes	1.65	1.32–2.01	.000	2.56	1.78–3.39	.000
Tested for HIV within the past year	No	1			1		
	Yes	1.42	1.08–1.88	.013	1.78	1.25–2.54	.001
HIV/STI knowledge score	0–4	1			1		
	5(full score)	1.22	0.98–1.52	.081	1.59	1.18–2.16	.003

OR, odds ratio; CI, confidence interval
